# NeglectARm3D: a robotic and virtual reality platform for the three-dimensional assessment of unilateral spatial neglect

**DOI:** 10.3389/frobt.2026.1944332

**Published:** 2026-09-09

**Authors:** Daniele Somma, Andrea Mannini, Chiara Pedrini, Francesca Cecchi, Egidio Falotico

**Affiliations:** 1 The BioRobotics Institute, Scuola Superiore Sant’Anna, Pontedera, Italy; 2 Department of Excellence in Robotics and AI, Scuola Superiore Sant’Anna, Pisa, Italy; 3 IRCCS Fondazione Don Carlo Gnocchi ETS, Firenze, Italy; 4 Department of Experimental and Clinical Medicine, University of Florence, Firenze, Italy

**Keywords:** 3D kinematics, human-robot interaction, robot-assisted assessment, stroke rehabilitation, unilateral spatial neglect, variable impedance control, virtual reality

## Abstract

Unilateral Spatial Neglect (USN) significantly impacts functional outcomes post-stroke, yet traditional assessment methods, relying predominantly on two-dimensional (2D) paper-and-pencil tasks, fail to capture the disorder’s complexity across three-dimensional (3D) space and, in particular, the role of depth. This paper introduces NeglectARm3D, a platform for the objective and repeatable 3D assessment of USN that integrates the precise kinematic and force measurement of a 7-DOF torque-controlled robot (Franka Research 3) with the immersive 3D stimulus presentation of a commercial head-mounted display (Meta Quest 3). A Cartesian variable impedance controller lets the participant reach freely toward randomized 3D targets (Phase 1, subject in charge), enabling the reconstruction of a volumetric exploration map, and delivers smooth haptic guidance toward neglected targets (Phase 2, robot in charge). As a first step toward clinical deployment, we evaluated the technical behavior, safety, and tolerability of the platform in twelve healthy adults performing thirty reaching trajectories (twenty in Phase 1, ten in Phase 2). The system preserved natural movement velocity ( 0.10±0.02 vs. 0.10±0.01 m/s), achieved large position error in free reaching ( 32.46±14.71 mm) *versus* precise robot-led convergence ( 3.01±0.43 mm) with reduced interaction force ( 4.96±0.84 vs. 2.59±0.32 N), and maintained physiological movement smoothness (SPARC −1.87±0.29 vs. −1.63±0.32 , benchmark −1.40 ). Usability was high (System Usability Scale 85.60±8.10 ), simulator sickness was low across all subscales, and all participants reported no pain and no perception of danger. These results support the technical feasibility and short-term tolerability of the integrated platform in healthy adults and justify subsequent clinical and psychometric evaluation in stroke survivors with USN.

## 1 Introduction

**FIGURE 1:**
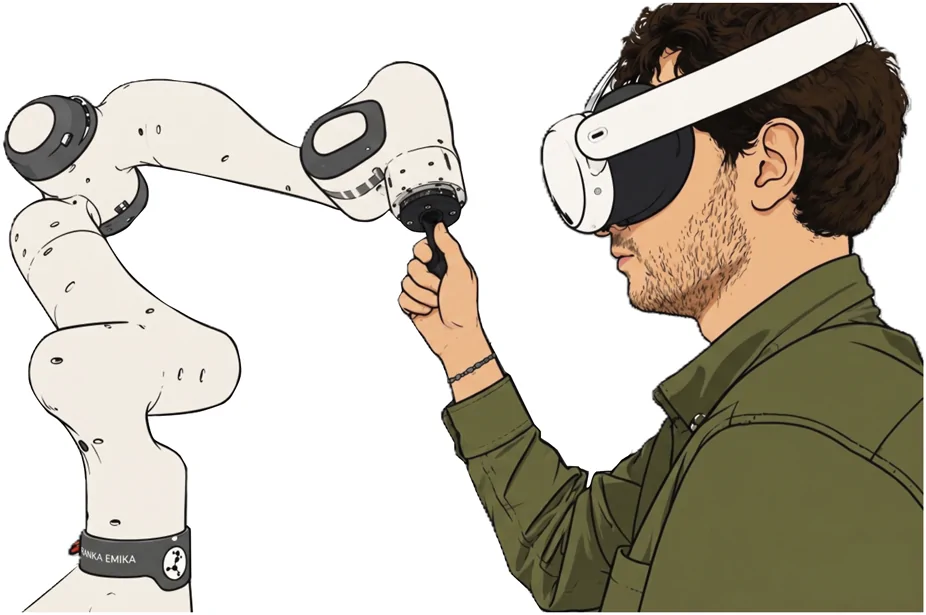
Experimental setup of NeglectARm3D. The participant wears a Meta Quest three head-mounted display and grasps a handle mounted at the flange of a Franka Research 3 7-DOF torque-controlled robot. The physical handle is co-located with a virtual cursor in the rendered 3D scene, providing congruent visuo-motor feedback while the robot records kinematics and interaction forces.

Unilateral Spatial Neglect (USN) is a failure to report, respond, or orient to stimuli in contralesional space that is not explained by primary sensory or motor deficits (Stone et al., 1991; Jacobs et al., 2012). This disabling condition typically follows unilateral brain damage, particularly in the right hemisphere, and is commonly associated with cerebral infarction or hemorrhage, affecting up to two-thirds of survivors with acute right-hemisphere stroke (Halligan and Marshall, 1991). USN markedly impairs rehabilitation and functional recovery and is associated with diminished quality of life (Corbetta et al., 2005). It is a heterogeneous disorder that may affect personal, peripersonal, and/or extrapersonal space (Pizzamiglio et al., 1989), and that can dissociate along several axes, spatial domain, frame of reference, and sensory modality (Jacobs et al., 2012), so that the deficit observed depends strongly on the spatial layout and demands of the task probing it (Bonato, 2012). In current clinical practice, assessment and treatment rely predominantly on two-dimensional (2D) representations of space, such as line-bisection and cancellation tasks (Bonato, 2012). These instruments provide robust, validated measures of spatial attention, but by construction they operate within a single fronto-parallel plane. They therefore offer limited ecological validity and only partially reflect the 3D spatial interactions that are critical for real-world functioning, where USN manifests across the volume of peripersonal and extrapersonal space. Crucially, behavior in near space can dissociate from behavior in far space (Pizzamiglio et al., 1989), and neglect can vary along the depth axis (Kageyama et al., 1994); yet depth remains a little-considered dimension in routine USN evaluation. The neural architecture of spatial attention provides a plausible substrate for these dissociations. Neglect reflects an imbalance within large-scale fronto-parietal attention networks, together with impaired re-orienting and arousal, rather than a simple sensory loss (Corbetta et al., 2005). The posterior parietal cortex, in particular, integrates visual information with the motor plans that act upon it, providing a neural locus where vision and reaching converge (Fattori et al., 2024). Because reaching toward a target at arm’s length necessarily engages depth, an assessment that couples controlled visual stimulation with active 3D reaching probes precisely this visuo-motor convergence, something a static, planar task cannot do. Virtual Reality (VR) and robotics offer complementary means to overcome these limitations (Cavedoni et al., 2022). VR can render controlled, multisensory stimuli throughout a volumetric environment and reproduce ecologically relevant scenarios, an advantage over planar tools. Robot-assisted arm training (RAT) offers repetitive upper-limb movement in multiple control modalities (assistive, assist-as-needed, resistive) and, importantly, objective measurement of kinematics and interaction forces (Basteris et al., 2014). Engaging the contralesional, often hemiparetic, arm in active movement, together with somatosensory stimulation of the affected side, has moreover been associated with reductions of visuospatial deficits in USN (Varalta et al., 2014; Park, 2021). This paper introduces NeglectARm3D, a hybrid platform that integrates an end-effector robot (Franka Research 3) with an immersive virtual reality headset (Meta Quest 3), shown in Figure 1. The system leverages the precise motor and force interaction of the robot within the expansive, multisensory 3D environment provided by VR. Its primary objective is to enable a quantifiable 3D mapping of spatial neglect with a critical focus on the role of depth. It is important to separate this long-term goal of the *platform*, characterizing neglect as a function of spatial position, including depth, from the goal of the *present study*, which is a preliminary validation in neurologically healthy adults. Taken together, three gaps in current practice motivate this work. First, 2D paper-and-pencil tests operate within a single fronto-parallel plane and cannot probe the depth dimension of neglect, even though behavior in near and far space can dissociate and neglect severity can vary with depth (Pizzamiglio et al., 1989; Kageyama et al., 1994). Second, although robotics offers objective, repeatable measurement of kinematics and interaction forces, it is used almost exclusively for treatment rather than assessment in the USN literature (Somma et al., 2026), so robot-based 3D evaluation of neglect remains essentially unexplored. Third, to the best of our knowledge no prior system couples a torque-controlled manipulator with immersive VR to characterize neglect as a function of 3D position throughout the reachable volume. NeglectARm3D is designed to close these three gaps at once. Accordingly, the contributions of this work are to:

1. Describe the technical architecture and operational mechanism of the NeglectARm3D system;
2. Validate the system’s technical feasibility, guidance precision, interaction safety, and usability in healthy subjects;
3. Establish normative, depth- and side-resolved reaching kinematics that serve both as a sensitivity check on the platform and as a reference baseline for the planned clinical study in stroke survivors with USN.

Because the same robot that measures free reaching can also physically guide the hand toward a stimulus, NeglectARm3D is conceived not only as a three-dimensional assessment tool but also as a potential rehabilitation device: much as a therapist points to an omitted target, the robot can actively bring the patient’s hand toward neglected locations, potentially engaging sensorimotor plasticity mechanisms distinct from purely visual cueing. The present study is a first technology validation of this dual-purpose platform in healthy adults.

### 1.1 Related work and state of the art

Although 3D technologies for USN are increasingly explored, they remain at an early, largely experimental stage, with substantial methodological heterogeneity and limited psychometric validation (Somma et al., 2026; Terruzzi et al., 2023). Currently, immersive and non-immersive VR are the most frequent technologies, whereas robotics appears in only a small minority of studies and almost exclusively for treatment rather than assessment (Somma et al., 2026), and even when immersive VR is combined with eye-tracking, more sensitive and ecologically valid methods are still called for to capture milder or atypical presentations of the disorder (Kaiser et al., 2022). Robot-based 3D *evaluation* of USN is therefore essentially unexplored, a gap a 3D, kinematics-based approach such as ours is positioned to address.

#### 1.1.1 Virtual reality technologies

VR promotes spatial recalibration across domains more effectively than 2D methods, integrating visual, motor, and attentional feedback (Cavedoni et al., 2022). It is valued for enabling precise control of visual stimuli in 3D space (Knobel et al., 2020), for an intuitive interface suitable for tele-rehabilitation (Morse et al., 2022), and for covering a wide movement range spanning personal to extrapersonal space (Perez-Marcos et al., 2023). Its main limitation is a limited capacity to provide motor cues, force feedback, or mechanical resistance: VR can show where a target is, but it cannot, on its own, measure the forces a patient exerts or physically guide the limb toward a neglected location. This capacity to engage far space was demonstrated early on with a head-mounted-display system that simulated a street-crossing task to train stroke patients with unilateral neglect (Kim et al., 2007), one of the first applications of immersive VR to engage extrapersonal space in a naturalistic, safety-critical scenario that could not be replicated with paper-and-pencil tools, and later adapted into a three-dimensional immersive virtual street-crossing program to assess extrapersonal neglect after stroke (Kim et al., 2010), showing that immersive VR can extend USN assessment beyond the near, peripersonal space that dominates conventional testing.

#### 1.1.2 Robotics in USN

Robotic interfaces complement VR exactly where it is weakest. They record kinematics and interaction forces objectively, can deliver assistive or resistive force fields adapted to the patient’s motor abilities, and engage the contralesional arm in active, repetitive movement, thereby limiting compensatory strategies (Basteris et al., 2014). Despite these advantages, robotics is rarely used for *assessment*, it appears almost exclusively in treatment protocols, partly because of mechanical complexity, cost, and space requirements (Somma et al., 2026). The few studies that paired robotics with USN, such as haptic virtual rehabilitation (Broeren et al., 2009) and robot-assisted arm training (Chen et al., 2021), together with an upper-limb rehabilitation robot shown to improve hemispatial neglect in stroke patients (Choi et al., 2016), were confined to planar or low-dimensional reaching within personal or peripersonal space, and none exploited the manipulator to characterize neglect along the depth axis. Despite that electromechanical and robotic training improves activities of daily living and arm function after stroke in general (Mehrholz et al., 2018); the well-established therapeutic role of these devices stands in contrast to the near-absence of robotic assessment tools specifically targeting USN, the gap this work addresses.

#### 1.1.3 The hybrid approach

The need for integrated, hybrid systems thus arises from the complementary limitations of the two modalities: VR’s lack of motor control and force feedback, and robotics’ limited spatial reach and multisensory richness (Somma et al., 2026). By integrating the precise motor and force capabilities of an end-effector robot with the expansive 3D environment of immersive VR, NeglectARm3D aims to provide quantifiable motor interaction across a wide spatial range, enabling a comprehensive assessment of neglect that includes the often-overlooked influence of depth. To the best of our knowledge, no prior system couples a torque-controlled manipulator with immersive VR to characterize neglect as a function of position throughout the reachable volume.

## 2 Materials and methods

### 2.1 System description: NeglectARm3D

#### 2.1.1 Hardware architecture

The platform comprises two main hardware components:

- Franka Research 3 (Franka Robotics, Munich), a 7-DOF robotic arm with joint-torque sensing, enabling force estimation and compliant interaction. It serves as the primary interface for active motor interaction, with a handle mounted at its flange that the participant grasps.
- Meta Quest 3 (Meta, Menlo Park), providing the immersive, multisensory 3D environment in which targets are presented. Its head-mounted display delivers full stereoscopic 3D immersion, supporting spatial recalibration through integrated visual and attentional feedback.

#### 2.1.2 Virtual environment and 3D mapping

**FIGURE 2:**
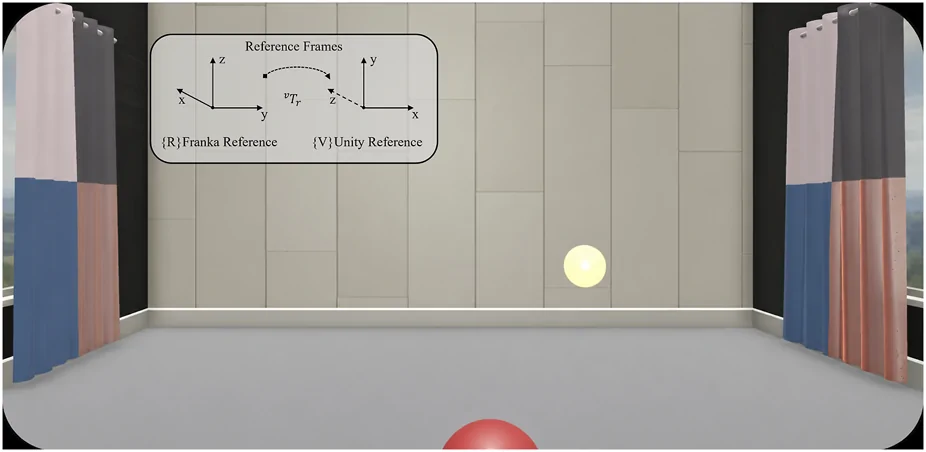
First-person view of the immersive virtual environment rendered on the head-mounted display. Spherical targets (here yellow) appear at randomized 3D positions across the reachable workspace, including different depths. The red sphere represents the position of the end-effector.

The virtual scene, shown in Figure 2, places the participant in a simple, low-distraction room in which spherical targets appear, one at a time, at randomized 3D positions distributed across the lateral, vertical, and depth extent of the reachable workspace. A fixed rigid transformation aligns the physical robot frame with the virtual world frame so that the end-effector is co-located with a virtual cursor; this alignment is essential to avoid visuo-proprioceptive conflict during the assessment. The virtual environment was developed in Unity3D, and communication with the robot relied on the Robot Operating System (ROS 1, Noetic): a middleware layer streams the robot state at 1 kHz, while the ROS–TCP Connector bridges the end-effector coordinates to the Unity rendering engine, which draws the environment and updates the display at 60 Hz. This decoupled architecture ensures that graphical rendering does not interfere with the high-frequency control loop required for stable haptics. Targets are rendered as spheres that change colour once reached, providing the participant with immediate visual feedback. For safety, only the manufacturer’s default Franka limits were enforced, together with a bounded operating workspace: the robot halted automatically whenever the end-effector left the predefined volume. By tracking the end-effector position and trajectory while the participant reaches toward targets distributed across the workspace, the system records the visuo-motor interaction and is designed to generate a quantifiable 3D map of spatial exploration, with explicit sampling of the depth ( x ) axis.

#### 2.1.3 Coordinate mapping and calibration

A critical requirement of any VR–robotic system is the alignment of the physical robot frame {R} and the virtual world frame {V} : a residual misalignment would induce a visuo-proprioceptive conflict able to confound the assessment. The end-effector position is expressed in the virtual frame through a rigid homogeneous transformation (Equation 1):

$$P_{V}{=}^{V}T_{R}\;P_{R},$$ (1)

where $P_{R}={\left[x,y,z,1\right]}^{⊤}$ is the end-effector position in the robot base frame ${\text{and }}^{V}T_{R}$ is a fixed rigid transformation that reorients the robot frame into the headset frame. It is applied online so that the rendered cursor and the physical handle remain co-located throughout the task. The transformation is a fixed rotation-only rigid alignment, determined once at system start-up.

#### 2.1.4 Control strategy

Physical interaction is governed by a Cartesian variable impedance controller. The commanded end-effector wrench is

$$F_{\mathrm{cmd}}=K\;\left({\bar{p}}_{eq}-{\bar{p}}_{ee}\right)+D\;\left({\dot{\bar{p}}}_{eq}-{\dot{\bar{p}}}_{ee}\right)+\Lambda \left(q\right)\;{\ddot{\bar{p}}}_{ee,des}+\mu \left(q,\dot{q}\right),$$ (2)

where ${\bar{p}}_{ee}$ and ${\dot{\bar{p}}}_{ee}$ are the measured end-effector position and velocity, ${\bar{p}}_{eq}$ the equilibrium (reference) position, and ${\ddot{\bar{p}}}_{ee,des}$ the desired end-effector acceleration. The first two terms realize the elastic and viscous behavior of the virtual impedance through the stiffness K and damping D matrices; Λ(q) provides inertial compensation based on the configuration-dependent Cartesian inertia, and $\mu \left(q,\dot{q}\right)$ compensates for the Coriolis, centrifugal, and gravitational dynamics of the manipulator (Hogan, 1985), consistent with variable-impedance formulations developed for redundant manipulators in physical human–robot interaction (Ficuciello et al., 2015). The same control law implements both operating regimes by modulating the Cartesian stiffness K : it was set to K=0 in Phase 1 and to K=80 N ${\text{m}}^{-1}$ in Phase 2, with the damping set to $D=2\sqrt{K}$ to approach the target without oscillation or overshoot. The stiffness value K=80N/m was selected empirically: several values were tested during pilot trials, and K=80N/m was the one that best reproduced a natural, human-like guided movement, avoiding over-damped motion and abrupt, jerky convergence to the target. This task-dependent modulation of stiffness between a free-reaching and a guided regime is consistent with variable-impedance strategies proposed for rehabilitation robots performing reaching tasks (Tang et al., 2022).

##### 2.1.4.1 Phase 1 – Subject in charge

With K=0 the elastic and viscous terms of Equation 2 vanish and the controller only compensates the manipulator’s own gravity, Coriolis, and inertial dynamics, rendering the robot essentially transparent: its intrinsic resistance to motion is close to zero and the participant moves the handle freely toward each target. The interaction force measured in this phase is therefore the force the participant actively applies to accelerate and steer the handle, not a resistance imposed by the device. The resulting trajectories sample the explored volume and, in the clinical use case, are intended to reconstruct a volumetric neglect map.

##### 2.1.4.2 Phase 2 – Robot in charge

In this phase the stiffness is raised ( K=80 N ${\text{m}}^{-1}$ ) so that the controller drives the end-effector toward the target with smooth, compliant motion and no abrupt force transients. To probe this regime in isolation, participants were explicitly instructed not to move toward the target but to relax and let the robot displace their hand. The phase thus characterizes the robot-led mode that, in patients, would assist reaching toward a neglected location, and it quantifies the forces exchanged when the device, rather than the user, leads the movement.

### 2.2 Assessment metrics

#### 2.2.1 Objective, robot-derived metrics

Unlike traditional pen-and-paper tests, which yield only a binary found/not-found score, NeglectARm3D captures the full kinematics and dynamics of reaching. For each trajectory we compute four complementary metrics:

- Average end-effector velocity [m/s], the mean speed along the trajectory, used to verify that natural movement dynamics are preserved.
- Position error [mm], the Euclidean distance d between the target $P_{\mathrm{target}}$ and the final hand position $P_{\mathrm{hand}}$ , $d=\|P_{\mathrm{target}}-P_{\mathrm{hand}}{\|}_{2}$ , quantifying reaching and guidance accuracy.
- Average interaction force [N], the mean magnitude of the physical force exchanged at the handle, estimated from the manipulator’s joint-torque sensing (external force/torque estimate) rather than measured with a dedicated external force/torque sensor, used as a proxy for effort and physical safety.
- Movement smoothness, quantified by the Spectral Arc Length (SPARC) (Equation 3) (Balasubramanian et al., 2015):

$$SPARC=-\;\int_{0}^{f_{c}}\sqrt{{\left(\frac{1}{f_{c}}\right)}^{2}+{\left(\frac{d\hat{V}\left(f\right)}{df}\right)}^{2}}\;df,$$ (3)

where $\hat{V}\left(f\right)$ is the normalized Fourier magnitude spectrum of the speed profile and $f_{c}$ a cutoff frequency. SPARC is negative, with values closer to zero indicating smoother movement; −1.40 is used as a reference benchmark for well-coordinated reaching. This value corresponds to the SPARC of the theoretical minimum-jerk velocity profile (Balasubramanian et al., 2015), computed with the cutoff frequency set at the point capturing 95% of the spectral amplitude.

For each trial, the active-movement window spans from motion onset to arrival at the target (Phase 1) or to the end of the guided motion (Phase 2). Prior to computing velocity, position error, and SPARC, the recorded end-effector position was low-pass filtered with a second-order Butterworth filter (15 Hz cutoff) to attenuate sensor noise while preserving the movement’s kinematic content. As a further summary of spatial coverage, the *explored volume* was computed, for each subject, as the volume of the three-dimensional convex hull enclosing the end-points of that subject’s Phase-1 reaching trajectories, and averaged across subjects.

#### 2.2.2 Subjective, questionnaire-based measures

Subjective experience was captured by four self-report instruments, administered after the reaching task: the System Usability Scale (SUS) (Brooke, 1996), a 10-item questionnaire scored on a 0–100 scale (acceptability threshold of 68); the Simulator Sickness Questionnaire (SSQ) (Kennedy et al., 1993), whose items are rated on a 0 (none) to 3 (severe) intensity scale; an *ad hoc* comfort and ergonomics questionnaire, with items rated on a 1–5 scale; and a set of *ad hoc* self-reported interaction-safety items. The comfort/ergonomics and interaction-safety items were developed *ad hoc* for this study, as no standardized instrument specifically addresses comfort and safety during combined robotic–VR interaction; they were designed to capture two aspects not covered by the SUS or the SSQ: the physical comfort of wearing the headset and holding the robot handle, and the participant’s subjective sense of safety and movement naturalness during robot-led guidance. The items were as follows.

Comfort and Ergonomics:

- How comfortable was wearing the headset for the entire duration of the session? [1–5]
- Did you feel excessive weight or pain on your face, neck, or head? [Yes/No]
- Was the robot handle (end-effector) comfortable to hold? [1–5]
- Did your arm feel excessively tired at the end of the exercise? [1–5]

Interaction and Safety:

- When the robot guided you, did the movement feel natural or “jerky”? [Natural/Jerky]
- Did you ever feel in danger or scared by the robot’s movements? [Yes/No]
- Did you notice any delays between your hand movement and what you saw in the headset? [Yes/No]
- Were the virtual targets easy to see and distinguish? [1–5]

### 2.3 Experimental validation

#### 2.3.1 Participants

Twelve healthy adults ( N=12 ; 8 men, 4 women; mean age 28.2±3.3 years, range 25–34) with no history of neurological or orthopedic impairment took part in the study. All participants performed the task with their dominant arm and had little to no prior experience with virtual reality. This study involved a technical and human-factors validation of the NeglectARm3D platform in neurologically healthy adult volunteers and did not involve patients or a clinical intervention. The research was conducted in accordance with the ethical principles of the Declaration of Helsinki. All participants were informed of the study procedures, risks, and their right to withdraw at any time, and provided informed consent prior to participation.

#### 2.3.2 Experimental protocol

After a familiarization period in which participants practiced moving the transparent robot to acclimatize to its dynamics, each participant performed thirty reaching trajectories toward randomized 3D targets distributed across the lateral, vertical, and depth extent of the reachable, peripersonal workspace: twenty with the subject in charge (Phase 1) and ten with the robot in charge (Phase 2). The unequal split reflects the different statistical demands of each phase. Phase 1 trials are subdivided by depth (Near/Mid/Far) (Pizzamiglio et al., 1989) and side for the analyses in Section 3, so twenty trials were needed for a stable per-subject mean in each bin. Phase 2 instead yields a single within-subject mean compared against Phase 1: ten trials were sufficient for this purpose while keeping session duration and fatigue contained. In Phase 1 participants reached freely toward each target; in Phase 2 they were instructed to remain passive and let the robot move their hand to the target. The study was performed in healthy subjects with the explicit goal of characterizing the technical behavior, safety, and tolerability of the platform prior to patient recruitment. The same targets were presented to all the subjects.

#### 2.3.3 Data analysis

For each participant and phase, we first computed a per-subject summary of every metric (the mean across that subject’s trajectories), yielding one paired value per subject in Phase 1 and Phase 2. All statistics were then performed on these per-subject values, so that the unit of analysis is the subject (N=12) , not the individual trajectory, avoiding pseudo-replication. Descriptive results are reported as mean ± standard deviation across subjects. Phase 1 characterizes the platform during free, subject-driven reaching, while Phase 2 characterizes the robot-driven guidance; the two phases were compared at the subject level using Wilcoxon signed-rank tests, with the paired effect size reported as Cohen’s $d_{z}$ . To characterize the spatial structure of free reaching, Phase 1 trials were grouped by target *depth* (tertiles of the reach-axis offset from the home position: Near/Mid/Far) and by lateral *side* (targets medial/lateral to the body midline). Depth effects were tested with the Friedman test (with Kendall’s W as effect size) and complemented by per-subject Spearman correlations between each metric and continuous depth; lateral symmetry was tested with Wilcoxon signed-rank tests across subjects. Given the small sample, non-parametric tests were used throughout; the reported Friedman p -values are uncorrected, and after Bonferroni correction for the four depth metrics movement time is the only one to approach significance $\left(p_{\text{Bonferroni}}=0.052\right)$ , only marginally above the α=0.05 threshold, and is further supported by its continuous Spearman correlation. Significance was set at α=0.05 . As all participants were neurologically healthy, these analyses are not diagnostic of neglect: they quantify the platform’s sensitivity across the workspace and provide normative reference values. SUS scores were averaged across participants and compared against the standard acceptability threshold; SSQ, comfort, and interaction-safety responses were summarized descriptively.

## 3 Results

### 3.1 3D workspace exploration

**FIGURE 3:**
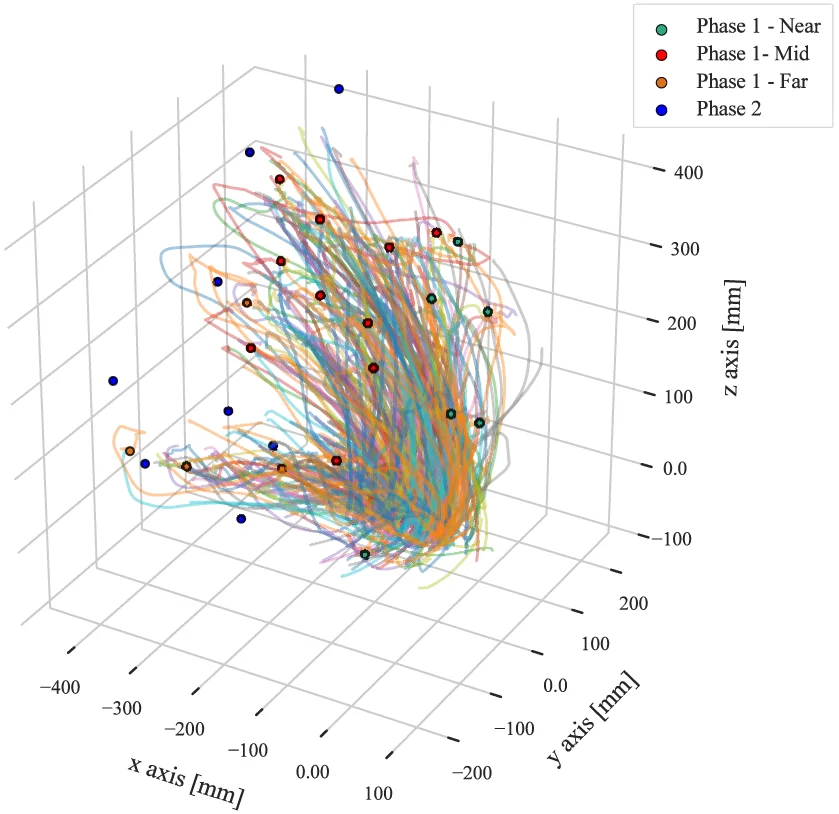
Three-dimensional end-effector trajectories of all twelve participants during the reaching task. Each colour corresponds to one subject; markers denote target positions. The trajectories sample the lateral, vertical, and depth extent of the reachable workspace.

Across the twelve participants, the recorded reaching trajectories sampled the 3D volume in front of the subject, spanning the lateral (y) , vertical (z) , and depth (x) directions (Figure 3). The coverage confirms that the platform can elicit and record reaching toward targets distributed throughout the reachable workspace, including at different depths, a prerequisite for the volumetric neglect mapping that is the clinical goal of the system. Targets were placed within a peripersonal-space box with target depth spanning 5–394 mm from the home position. Targets were grouped by depth (the absolute offset from the home position along the reach axis, x ) into three equal-frequency bins (tertiles): Near (5–146 mm), Mid (146–257 mm), and Far (257–394 mm). Per subject, the convex hull of the Phase-1 reaching end-points enclosed a mean explored volume of $1.25\times 10^{7}\pm 2.6\times 10^{6}$ ${\text{mm}}^{3}$ , confirming that participants consistently covered the target volume.

### 3.2 Reaching Performance and Interaction

**FIGURE 4:**
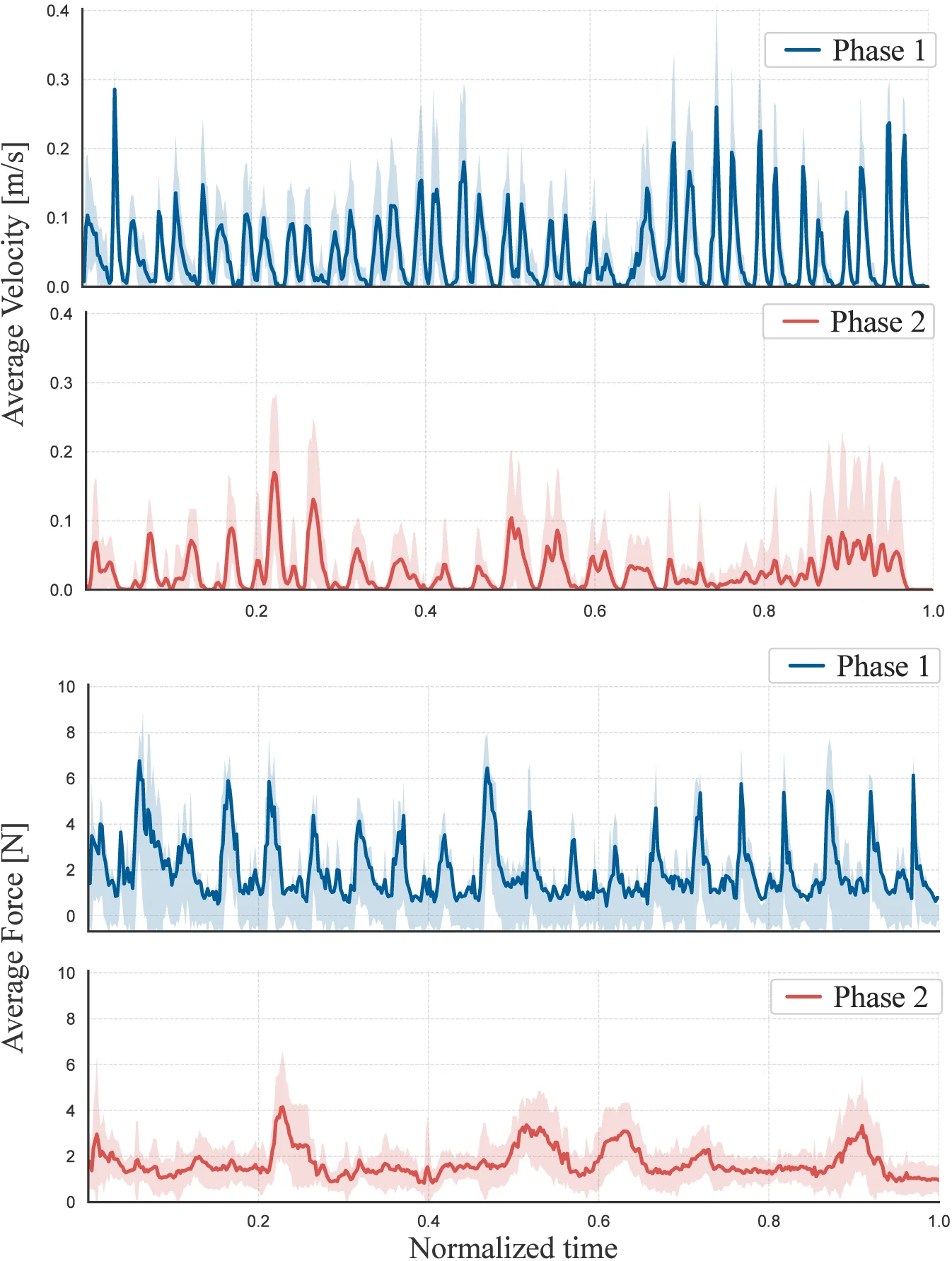
End-effector velocity (top two panels) and interaction force (bottom two panels) over normalized time. Solid lines are the mean across subjects and trajectories, shaded areas the standard deviation; blue = Phase 1 (subject in charge), red = Phase 2 (robot in charge).

Table 1 summarizes the quantitative metrics, and Figure 4 shows the corresponding velocity and force profiles over normalized time. The average end-effector velocity was essentially identical in the two phases ( 0.10±0.02 vs. 0.10±0.01 m/s), confirming that natural movement speed was preserved both during free reaching and under robot guidance; the velocity profiles show that the characteristic reaching peaks of Phase 1 are preserved in Phase 2, while their amplitude is regularized. Position error differed markedly: 32.46±14.71 mm during free reaching, reflecting the natural variability of human reaching toward 3D targets, *versus* 3.01±0.43 mm under guidance, about an order of magnitude smaller, indicating precise convergence of the guided trajectory to the target. The average interaction force decreased from 4.96±0.84 N (Phase 1) to 2.59±0.32 N (Phase 2); the force profiles confirm that the robot leads the movement with lower and smoother forces. This indicates low-effort physical interaction in this healthy-adult cohort; interaction safety itself was assessed independently through the self-reported outcomes in Section 3.7, rather than inferred from force magnitude alone. Movement smoothness lay within the physiological range in both phases ( −1.87±0.29 and −1.63±0.32 ), with the guided trajectories closer to the −1.40 benchmark, indicating that the compliant controller attenuates the velocity fluctuations of free reaching without introducing jerky corrections. Paired subject-level comparisons supported these observations: velocity did not differ between phases ( p=0.47 ; $d_{z}=0.06$ ), whereas the interaction force was substantially lower under robot guidance ( p<0.001 ; $d_{z}=2.60$ ) and the movement was significantly smoother (SPARC, p=0.02 ; $d_{z}=0.75$ ). The velocity and force profiles (Figure 4) make the difference between the two regimes explicit. In Phase 1 the velocity trace is strongly intermittent, exhibiting the repeated accelerate–decelerate peaks typical of visually guided human reaching, while the interaction force fluctuates around a higher mean as the participant actively drives the handle. In Phase 2 both signals become more regular: the guided velocity preserves a comparable average but with attenuated peaks, and the force settles onto a lower, smoother envelope. This pattern is consistent with the SPARC values reported in Table 1 and confirms that the controller reshapes the dynamics of the movement, reducing peak effort and variability, without suppressing the participant’s natural timing.

**TABLE 1:** Quantitative performance metrics (mean ± SD).

Metric	Phase 1	Phase 2
	(Subject in charge)	(Robot in charge)
Average velocity [m/s]	0.10±0.02	0.10±0.01
Position error [mm]	32.46±14.71	3.01±0.43
Average force [N]	4.96±0.84	2.59±0.32
SPARC [-]	−1.87±0.29	−1.63±0.32

### 3.3 Normative depth- and side-resolved kinematics

**FIGURE 5:**
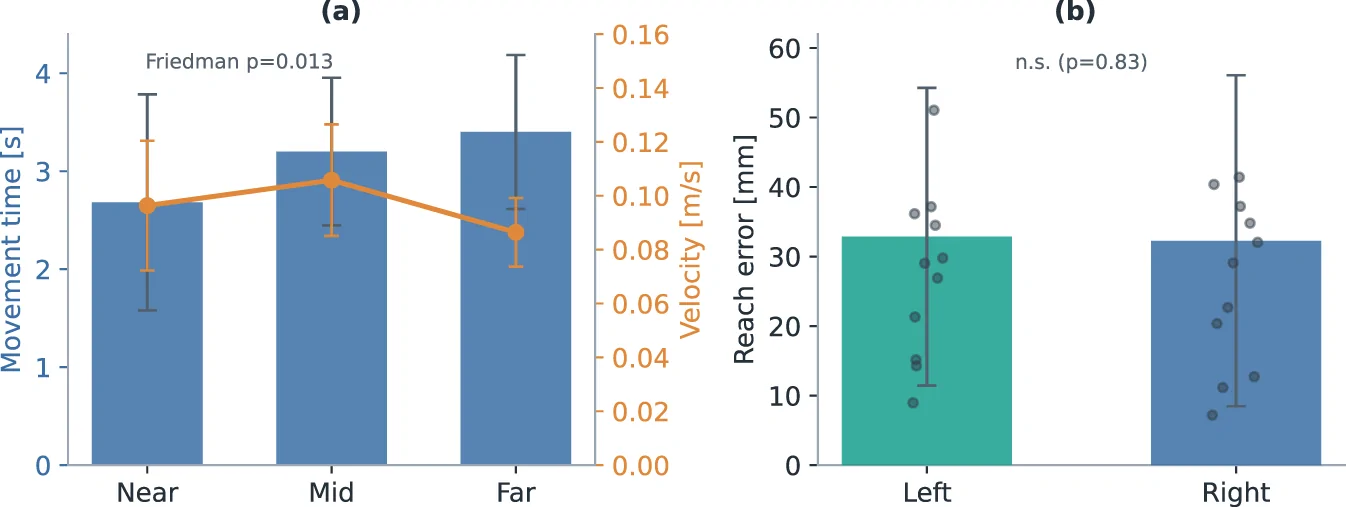
Normative Phase-1 kinematics. **(A)** Movement time (bars) and velocity (line) across target depth, showing the expected slowing with distance. **(B)** Reach error in the two lateral hemispaces; the difference is not significant, confirming left/right symmetry of accuracy in healthy subjects. Error bars are SD; dots in **(B)** are individual subjects.

To characterize how the platform’s metrics vary across the workspace during free reaching (Phase 1), we verified the system’s sensitivity to the depth and lateral dimensions (Table 2; Figure 5). Along the depth axis, movement time increased systematically from near to far targets ( 2.68→3.40 s; Friedman p=0.013 , W=0.36 ). Velocity and smoothness showed weaker, non-significant gradients in the same direction. Crucially for an instrument intended to detect lateralized deficits, reaching accuracy was laterally symmetric: the reach error did not differ between the medial and lateral hemispaces (32.90 vs. 32.30 mm; p=0.85 ), establishing the expected normative baseline of left/right symmetry in healthy adults. A modest right-side advantage in velocity and movement time was also observed, but it is most plausibly attributable to right-hand dominance and to the unbalanced lateral distribution of the fixed target set, rather than to a neural asymmetry, and is therefore reported with caution.

**TABLE 2:** Phase-1 (free reaching) metrics by target depth (mean ± SD across 12 subjects; Near/Mid/Far = tertiles of distance from the body). The p -value is from the Friedman test (uncorrected); after Bonferroni correction for the four metrics, Move time is the only one to approach significance $\left(p_{\text{Bonferroni}}=0.052\right)$ , only marginally above the α=0.05 threshold.

Metric	Near	Mid	Far	$p_{\text{Friedman}}$
Move time [s]	2.68±1.10	3.20±0.75	3.40±0.79	0.013
Velocity [m/s]	0.10±0.02	0.11±0.02	0.09±0.01	0.05
Reach err [mm]	28.40±14.51	30.82±19.34	37.80±39.41	0.26
SPARC [-]	−2.00±0.76	−1.74±0.14	−1.84±0.20	0.08

### 3.4 Usability

**FIGURE 6:**
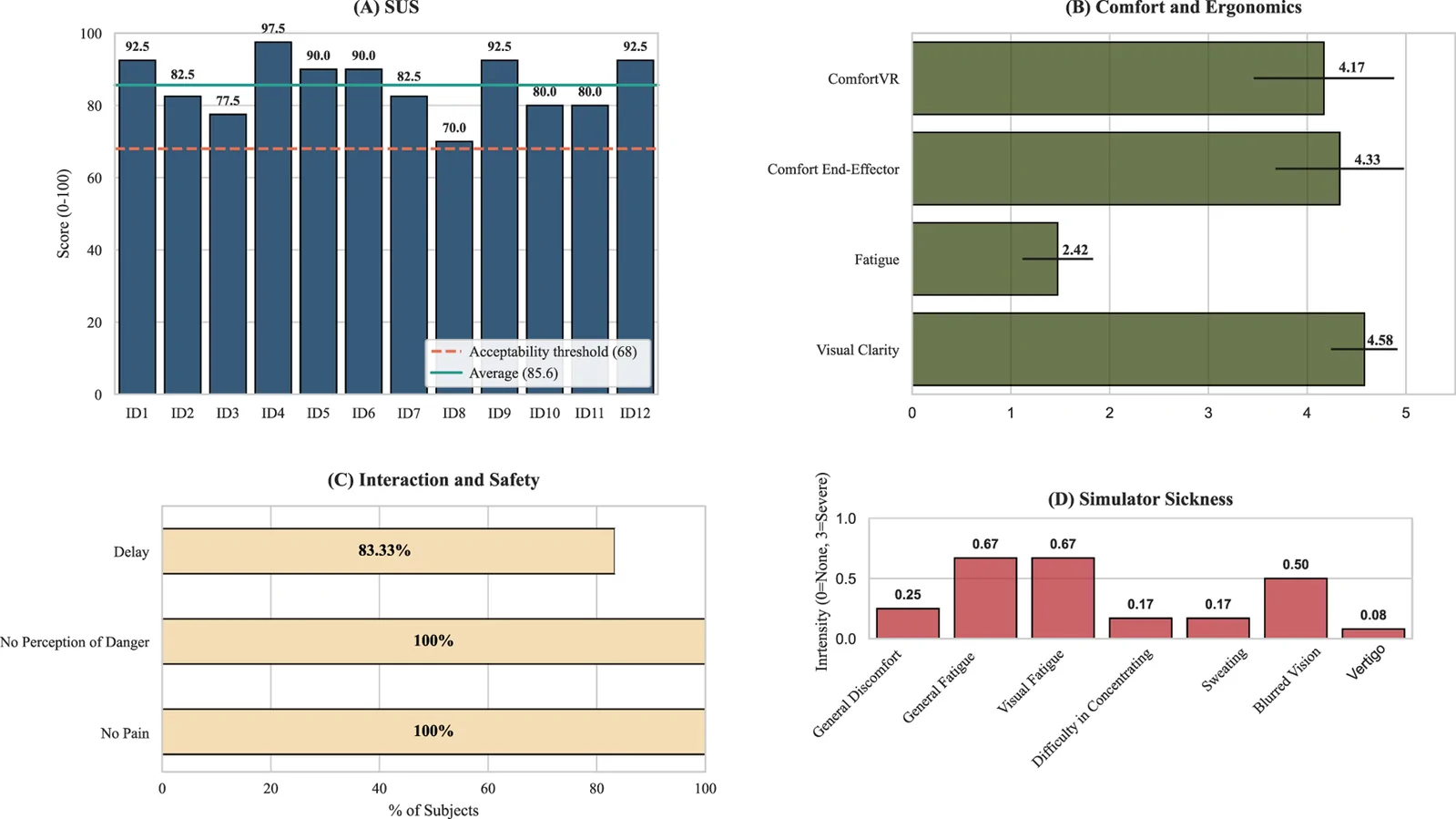
Self-report outcomes (N=12) . **(A)** Per-participant SUS scores (dashed: acceptability threshold 68; solid: mean 85.60). **(B)** Comfort and ergonomics ratings (1–5). **(C)** Interaction and safety (% of subjects). **(D)** Simulator Sickness Questionnaire item intensities (0 = none, 3 = severe).

SUS (Brooke, 1996) score averaged 85.60±8.10 (Figure 6A), placing the system in the upper, “excellent” band of usability. All twelve participants scored above the standard acceptability threshold of 68 (minimum 70.00, maximum 97.50), indicating that, despite requiring the user to wear a head-mounted display while operating a 7-axis manipulator, the platform was consistently perceived as easy to use.

### 3.5 Tolerability

Cybersickness was low across all SSQ subscales (Kennedy et al., 1993) (Figure 6B): on a 0 (none) to 3 (severe) scale, the highest mean intensities were general and visual fatigue (0.67), followed by blurred vision (0.50); general discomfort, difficulty concentrating, sweating, and vertigo all remained below 0.30. No participant reported symptoms that interrupted the session. The near-absence of cybersickness is notable, since simulator sickness is a recognized barrier to the clinical adoption of immersive VR and is sensitive to visuo-motor latency and sensory conflict.

### 3.6 Comfort and ergonomics

On the *ad hoc* comfort questionnaire (Figure 6C), participants rated visual clarity highest (4.58 out of 5), followed by the comfort of the end-effector handle (4.33) and of the VR headset (4.17); perceived fatigue was comparatively low (2.42). The high comfort and visual-clarity ratings, together with the low fatigue, indicate that prolonged interaction with the dual-device setup is tolerable.

### 3.7 Interaction and safety

Self-reported interaction safety was favorable (Figure 6D): all participants (100%) reported no pain and no perception of danger during the interaction, and 83.33% reported no perceptible delay between their movement and the system’s response. These results corroborate the low interaction forces measured in Phase 2 and support the short-term tolerability of the interaction under the tested experimental conditions.

## 4 Discussion

This study evaluated NeglectARm3D, a platform fusing immersive VR with a torque-controlled robot, in a healthy-subject pretest. The aim was not to detect neglect, healthy participants have none, but to verify that the integrated system behaves as intended, interacts safely, is well tolerated, and yields the normative kinematics needed to interpret future patient data. The results are discussed in that light.

### 4.1 Principal findings

Three results from the Reaching Performance and Interaction subsection (Table 1) are, taken together, the main technical outcome of this study. *First*, the guided mode (Phase 2) reproduced free-reaching velocity (no significant difference; Wilcoxon signed-rank, p=0.47 , $d_{z}=0.06$ ) while improving smoothness (Wilcoxon, p=0.02 , $d_{z}=0.75$ , closer to the −1.40 benchmark). This is notable because participants stayed passive in Phase 2: rather than imposing an arbitrary trajectory, the controller reproduced a naturally paced reach and slightly attenuated its velocity fluctuations. The two regimes follow directly from the control design, a transparent K=0 mode *versus* a guiding K=80 N ${\text{m}}^{-1}$ mode, and show that the device measures movement without distorting its timing. *Second*, guidance required substantially less force than free reaching (the largest effect observed, Wilcoxon signed-rank, p<0.001 , $d_{z}=2.60$ , consistent across all twelve subjects). Because the robot is transparent in Phase 1, the force measured there is the force the participant actively applies to move the handle, not a resistance imposed by the device; the lower Phase 2 value is the modest force exchanged when the user relaxes and the robot leads. This force-level quantification is exactly the information a VR-only system cannot provide, and the low effort is the property a device physically coupled to a fragile patient’s hand must guarantee, corroborated by the self-reported absence of pain or perceived danger (Section 3.7). *Third*, usability was high (SUS 85.60±8.10 , above the acceptability threshold for every participant) and cybersickness was minimal, together clearing the tolerability bar for a patient study.

### 4.2 Positioning relative to the state of the art

The quantities reported here, interaction forces in newtons, depth-resolved movement times, millimetric side-resolved accuracy, and a $1.25\times 10^{7}$ ${\text{mm}}^{3}$ explored volume: the platform provides measurements that are generally unavailable from conventional paper-and-pencil assessments and VR-only implementations. The few robotic USN efforts in the state of the art are planar or therapeutic (Varalta et al., 2014; Park, 2021; Broeren et al., 2009; Chen et al., 2021; Fordell et al., 2016), while immersive VR offers ecological 3D stimulation but cannot quantify reaching forces or deliver calibrated guidance (Cavedoni et al., 2022). NeglectARm3D differs on three axes at once, assessment rather than treatment, a 3D volume that samples depth, and force-level sensing and guidance. Beyond adding dimensions, recording graded kinematics and kinetics instead of an omission count changes the nature of the measurement. Mechanically, coupling a depth-resolved reach to a visual target engages the fronto-parietal and posterior-parietal visuomotor circuits implicated in neglect (Corbetta et al., 2005; Fattori et al., 2024), and independently controlling depth and body-relative position could begin to separate near/far and egocentric/allocentric components (Pizzamiglio et al., 1989; Kageyama et al., 1994). Finally, the same controller that measures in Phase 1 delivers, in Phase 2, the low-force (2.59 N) smooth guidance documented above; in patients this congruent visual–proprioceptive–motor episode is the kind of limb activation associated with transient reductions of visuospatial deficits (Varalta et al., 2014; Park, 2021), making the platform a candidate for a combined assessment–treatment pipeline rather than a single-purpose tool.

### 4.3 Depth and side-resolved kinematics

Reach error did not differ between the two hemispaces (32.90 vs. 32.30 mm; Wilcoxon signed-rank, p=0.85 ). Since neglect is a lateralized departure from symmetry, this 0.60 mm, non-significant difference is precisely the normative reference against which a contralesional deficit would be flagged in patients, a graded, side-resolved accuracy baseline that binary 2D omission tests cannot supply. The modest right-side advantage in velocity and movement time is confounded by right-hand dominance and by the unbalanced lateral placement of the fixed target set, and is therefore not interpreted as a neural asymmetry. *The recorded metrics are distance-sensitive.* Movement time increased monotonically with target depth ( 2.68→3.40 s; Friedman p=0.013 , Spearman $\bar{r}=+0.25$ , p=0.005 ), while velocity and smoothness showed no monotonic trend. However, in the present layout deeper targets are also farther from the home position, so depth and reach distance are confounded: the observed increase in movement time cannot be attributed to depth specifically.

### 4.4 Limitations

The study was conducted exclusively in a small sample of healthy adults, so it demonstrates feasibility and tolerability but says nothing about detecting, localizing, or quantifying neglect, which only a USN patient study can establish; accordingly the depth- and side-resolved results are normative, not diagnostic. Depth and reach distance were not dissociated in the present target layout, so depth-specific effects, as opposed to distance-sensitivity, cannot be isolated from the current data. Targets covered only the personal region, so far-space behavior was not probed. The volumetric neglect map motivating Phase 1 was used here to characterize device behavior, not yet validated as a clinical output, and the metrics still require formal psychometric evaluation; the absence of a 2D comparison condition also leaves the incremental value of the 3D approach to be demonstrated. Reliability and responsiveness, found to be under-reported for 3D assessment tools (Somma et al., 2026), remain unknown from a single session.

### 4.5 Future work

The natural next step is a study in stroke survivors with USN, designed from the outset for psychometric evaluation. It should (i) define and validate a quantitative 3D neglect map from the Phase 1 trajectories, with explicit analysis of performance *versus* depth; (ii) establish concurrent validity against the Behavioural Inattention Test and the Catherine Bergego Scale and quantify the incremental information from the depth dimension; (iii) include repeated measurements to estimate reliability, responsiveness, and minimal clinically important differences; and (iv) test whether Phase 2 guidance carries therapeutic value through limb activation. Larger, pre-registered protocols would further reduce the methodological heterogeneity that limits comparison across this field (Somma et al., 2026).

### 4.6 Conclusion

This paper presented NeglectARm3D, a platform that fuses a torque-controlled robot with immersive VR for the three-dimensional, depth-aware assessment of Unilateral Spatial Neglect. A feasibility study in twelve healthy adults showed that the platform preserves natural movement velocity, delivers accurate and low-force guidance, maintains physiological movement smoothness, and is rated as highly usable with minimal simulator sickness and no perceived pain or danger. The present study thus represents an engineering and human-factors validation, not a clinical validation of USN assessment. By using a robotic end-effector as a calibrated 3D probe within an immersive virtual environment, the system nonetheless addresses a concrete gap in the state of the art and provides the methodological foundation for the planned clinical study, in which the diagnostic value of a depth-resolved, robot-based neglect map will be tested against standard clinical scales.

## Data Availability

The raw data supporting the conclusions of this article will be made available by the authors, without undue reservation.
